# Risk factors for post-stroke depression in patients with mild and moderate strokes

**DOI:** 10.1097/MD.0000000000034157

**Published:** 2023-06-30

**Authors:** Wenxiang Liao, Danlei Chen, Jing Wu, Kaixiang Liu, Junlin Feng, Hao Li, Jingzi Jiang

**Affiliations:** a Neurology Department, Affiliated Hospital of Guilin Medical University, Guilin, Guangxi, China; b Geriatrics Department, Affiliated Hospital of Guilin Medical University, Guilin, Guangxi, China; c Neurology Department, Graduate College of Guilin Medical University, Guilin, Guangxi, China.

**Keywords:** activities of daily living, negative life event, post-stroke depression, social support, stroke frequency

## Abstract

To determine the possible risk factors for post-stroke depression in patients with mild and moderate acute strokes. A cross-sectional descriptive study was conducted involving 129 patients with mild and moderate acute strokes. The patients were divided into post-stroke depression and non-depressed stroke groups according to the Hamilton Depression Rating Scale for Depression-17 item and Patient Health Questionnaire-9 item assessments. All participants were evaluated based on clinical characteristics and a battery of scales. Patients with post-stroke depression had an increased stroke frequency, severe stroke symptoms and poor performance in activities of daily living (ADL), cognitive function, sleep quality, interest in pleasurable activities, negative life events, and utilization of social support compared to stroke patients without depression. The Negative Life Event Scale (LES) score was significantly and independently associated with an increased probability of depression in stroke patients. Negative life events were shown to be independently associated with the incidence of depression in patients with mild and moderate acute strokes, likely mediating the influence of other predictors of depression, such as a history of stroke, decreased ADL ability, and utilization of support.

## 1. Introduction

Stroke is the most common neurologic disease and the third-leading cause of disability-adjusted life-years worldwide.^[1]^ The onset of depression is a frequent complication and neuropsychiatric consequence of stroke, which may adversely affect recovery. Negative recovery outcomes include unsatisfactory rehabilitation, decreased quality of life, reduced level of community participation, and an increased risk of mortality.^[2–5]^ Prior studies have estimated that the prevalence of post-stroke depression (PSD) is 18% to 33%,^[6,7]^ and the cumulative incidence of PSD is 39% to 52% within 5 years of a stroke.^[8]^ Notably, PSD is underdiagnosed and undertreated despite the high prevalence.^[9]^

Various studies have confirmed and replicated the risk factors and predictors of PSD, which are comprised of several stroke features, including severity and lesion site.^[10–13]^ Moreover, the risk factors for PSD are similar to the risk factors for major depressive disorder (MDD), and include low social support and exposure to stressful life events^[14,15]^ or stroke (age and diabetes mellitus).^[13,16]^ Evidence in support of the association between these factors and PSD, although still a matter of debate, suggests that stoke severity and lower social support are common risk factors for depression after a stroke. The PSD risk factors related to stroke severity include an increased cerebral infarction lesion volume,^[10]^ multiple acute cerebral infarcts,^[11]^ and severe disability.^[12]^ Patients with a severe disabling stroke have a 7.9-fold risk of developing PSD than patients with a mild disabling stroke.^[12]^ Previous studies have confirmed that poor social support is associated with developing depressive symptoms consistent with MDD.^[17–19]^ Moreover, lower social support may impede symptom improvement in patients with MDD^[20]^ and predict MDD recurrence.^[21]^ A review in 2020 suggested that a lack of social support is associated with a higher risk of depression after a stroke.^[22]^ These results illustrate the independent correlation between social support and depressive symptoms. In addition, cerebral infarction lesions in the basal ganglia^[11,23]^ and left hemisphere^[6]^ have been shown to impact the incidence of depression after a stroke. In contrast, Ibrahimagic et al^[24]^ was of the opinion that the location of a stroke lesion is not associated with PSD. Several studies have concluded that gender, age, history of stroke, aphasia and diabetes mellitus may be independent risk factors for PSD;^[13,16,25]^ however, these results are mixed because not all of the studies replicated the conclusions due to variations in study design.

Despite an abundance of studies on clinical risk factors for PSD, the precise influence of a stroke on mood is controversial. In attempting to improve recovery outcomes for stroke patients, it is essential to accurately identify the risk factors for PSD. The present study determined the prevalence of PSD and identified the possible risk factors for PSD in patients with mild and moderate acute strokes.

## 2. Methods

### 2.1. Participants

A total of 129 patients with acute ischemic stroke participated in the present study. These patients were recruited as inpatients from the Neurology Department of The Affiliated Hospital of Guilin Medical University (Guilin, China). All the participants were administered the Hamilton Depression Rating Scale for Depression-17 item (HAMD-17) and Patient Health Questionnaire-9 item (PHQ-9) assessments. The patients were then divided into PSD (HAMD-17 scores ≥ 8 and PHQ-9 scores ≥ 5) and non-depressed stroke (NDS) groups based on the HAMD-17 and PHQ-9 scores. Stroke patients who met the following criteria were recruited: 40 to 80 years of age; ischemic stroke diagnosis within 7 days of stroke onset according to clinical and cranial computed tomography scan/magnetic resonance imaging findings; and mild or moderate stroke (National Institutes of Health Stroke Scale [NIHSS] <16). The exclusion criteria for all the participants were as follows: a history of any psychiatric diagnosis before stroke, such as schizophrenia and depression; a history of other neurologic diseases, such as epilepsy and Parkinson disease; aphasia (due to an inability to complete the scale assessment); and other severe medical diseases, such as myocardial infarction and end-stage renal failure. All participants provided written informed consent, and the study protocol was approved by the Hospital Ethical Committee for clinical research of Affiliated Hospital of Guilin Medical University in accordance with the Declaration of Helsinki.

### 2.2. Clinical evaluation and scales assessment

Demographic and clinical data were obtained from evaluations performed by highly-trained research staff and included the following variables: gender; age; years of education; present stroke lesion site; homocysteine, triglyceride, and low-density lipoprotein-cholesterol levels; medical history; cigarette smoking; and alcohol consumption. All participants were administered a battery of instruments, including the NIHSS, HAMD-17, PHQ-9, Mini-mental Status Examination, Activity of Daily Living (ADL), Pittsburgh Sleep Quality Index (PSQI), Snaith-Hamilton Pleasure Scale (SHAPS), Life Event Scale (LES) and Social Support Rating Scale.

### 2.3. Data analysis

Statistical Product and Service Solutions (SPSS, Version 23.0) was used for statistical analyses. Categorical variables are presented as the number and analyzed using a chi-square test. Other measurement data are shown as medians (quartiles) and analyzed using the Mann-Whitney test. Binary logistic regression analysis was used for risk factors that differed between the NDS and PSD groups.

## 3. Results

A total of 129 acute stroke patients were included in the current study, 46 (35.7%) of whom had depression. There were significant associations between depression and clinical characteristics, including gender, history of a stroke, and stroke symptom severity; the ADL, mini-mental status examination, PSQI, and SHAPS scores, a negative LES, and social support rating scale-utilization of support scores. Specifically, stroke patients with the following characteristics were more likely to be depressed: female gender, increased stroke frequency, severe stroke symptoms, decreased ADL ability, cognitive deficits, poor sleep quality, lack of interest in pleasurable activities, increased frequency of negative life events, and poorer utilization of support. The detailed data are presented in Tables 1 and 2.

**Table 1:** Clinical characteristics compared between NDS and PSD groups by chi-square test.

	NDS (n = 83)	PSD (n = 46)	χ^2^	*P*
Gender (male/female)	64/19	28/18	3.82	.05
Lesion side (left/right/both)	35/36/12	19/20/7	0.02	.99
Lesion site (cortex and sub-cortex/basal ganglia/posterior circulation/multiple foci)	21/25/24/13	10/14/13/9	0.43	.94
History of hypertension (yes/no)	61/22	38/8	1.38	.24
History of diabetes (yes/no)	21/62	16/30	1.30	.25
History of stroke (yes/no)	14/69	17/29	6.54	.01
Cigarette smoking (yes/no)	39/44	18/28	0.74	.39
Alcohol use (yes/no)	40/43	22/24	<0.01	.97

NDS = non-depressed stroke, PSD = post-stroke depression.

**Table 2:** Clinical characteristics and factors associated with depression in the NDS and PSD groups by the Mann-Whitney test.

	NDS (n = 83) [M (P25, P75)]	PSD (n = 46) [M (P25, P75)]	Z	*P*
Age (yr)	65.0 (57.0, 70.0)	66.0 (57.0,70.0)	−0.52	.60
Educational level	9.0 (6.0, 9.0)	7.0 (4.8, 12.0)	−1.20	.23
NIHSS scores	2.0 (1.0, 3.0)	3.0 (2.0, 4.0)	−2.95	<.01
Homocysteine level	16.6 (12.5, 20.4)	15.7 (11.9, 18.9)	−1.11	.27
TG	1.5 (1.0, 2.2)	1.5 (1.1, 2.2)	−0.25	.80
LDL-C	2.7 (2.2, 3.3)	2.4 (2.1, 3.1)	−1.79	.07
ADL	20.0 (20.0, 30.0)	38.0 (20.8, 63.3)	−4.80	<.001
MMSE	23.0 (21.0, 26.0)	21.5 (18.0, 23.0)	−3.21	<.01
HAMD-17	2.0 (0.0, 4.0)	17.0 (12.8, 22.3)	−9.36	<.001
PHQ-9	1.0 (0.0, 2.0)	16.0 (11.0, 19.3)	−9.44	<.001
PSQI	2.0 (1.0, 5.0)	10.0 (6.0,14.0)	−7.52	<.001
SHAPS	0.0 (0.0, 2.0)	5.0 (2.8, 7.3)	−7.39	<.001
Negative LES	2.0 (0.0, 8.0)	24.0 (16.0, 32.0)	−7.49	<.001
SSRS-total	41.0 (36.0, 45.0)	39.0 (34.8, 43.0)	−1.38	.17
SSRS-objective	8.0 (8.0, 11.0)	8.0 (7.0, 10.5)	−0.18	.86
SSRS-subjective	24.0 (22.0, 27.0)	23.0 (19.8, 26.3)	−0.94	.35
SSRS-utilization of support	7.0 (6.0, 9.0)	7.0 (5.0, 8.0)	−2.25	.02

ADL = Activity of Daily Living, HAMD-17 = Hamilton Depression Rating Scale for Depression-17 item, LDL-C = Low-density lipoprotein-cholesterol, LES = Life Event Scale, MMSE = Mini-mental Status Examination, NDS = non-depressed stroke, NIHSS = National Institutes of Health Stroke Scale, PHQ-9 = Patient Health Questionnaire-9-item, PSD = post-stroke depression, PSQI = Pittsburgh Sleep Quality Index, SHAPS = Snaith-Hamilton Pleasure Scale, SSRS = Social Support Rating Scale, TG = Triglyceride.

To determine the independent risk factors associated with PSD, binary logistic regression analysis was used. The negative LES, PSQI, and SHAPS scores were significantly and independently associated with PSD (Table 3). Because the HAMD content included sleep quality and a lack of interest in pleasurable activities, PSQI and SHAPS were considered part of the HAMD; these confounding factors could affect the logistic regression model. By building the model without PSQI and SHAPS, a negative LES score was also significantly associated with an increased probability of depression (Table 4). Thus, stroke patients with a negative LES > 32 were 226-fold more likely to develop depression than patients with a negative LES < 8. Of note, when the effect of negative life events were removed (Table 5), patients who had a history of stroke were at increased risk for PSD compared to patients who did not have a history of stroke (odd ratio = 2.88, 95% confidence interval: 1.06–7.83, *P* = .039). Specifically, stroke patients with poor ADL ability were more likely to have PSD. Better utilization of support was significantly associated with a lower probability of PSD (odd ratio = 0.79, 95% confidence interval: 0.62–0.99, *P* = .039).

**Table 3:** Binary logistic regression analysis of risk factors for PSD (Model I).

	OR	95% CI	*P*
Gender			
Male	1		
Female	1.82	0.31–10.65	.51
History of stroke			
No	1		
Yes	2.16	0.29–16.32	.46
Stroke symptom severity			
Mild (NIHSS score: 0–4)	1		
Moderate (NIHSS score: 5–15)	0.37	0.03–4.87	.45
MMSE	0.92	0.73–1.16	.50
ADL			.42
Score = 20	1		
Score:21–40	2.07	0.35–12.12	.42
Score: 41–60	4.30	0.36–50.85	.25
Score: 61–80	10.71	0.54–213.20	.12
SSRS-utilization of support	0.88	0.57–1.35	.552
Negative LES			<.01
Score: 0–8	1		
Score: 9–32	24.44	2.99–199.56	<.01
Score: >32	43.53	1.67–1132.01	.02
PSQI	1.44	1.15–1.80	<.01
SHAPS	1.35	1.01–1.79	.04
Constant	0.017		.24

In this model, method = enter, *P* = .777 in Hosmer and Lemeshow test.

ADL = Activity of Daily Living, CI = confidence interval, LES = Life Event Scale, MMSE = Mini-mental Status Examination, NIHSS = National Institutes of Health Stroke Scale, OR = odd ratio, PSQI = Pittsburgh Sleep Quality Index, SHAPS = Snaith-Hamilton Pleasure Scale, SSRS = Social Support Rating Scale.

**Table 4:** Binary logistic regression analysis of risk factors for PSD (Model II).

	OR	95% CI	*P*
Gender			
Male	1		
Female	1.47	0.41–5.31	.56
History of stroke			
No	1		
Yes	2.80	0.59–13.27	.20
Stroke symptom severity			
Mild (NIHSS score: 0–4)	1		
Moderate (NIHSS score: 5–15)	0.24	0.03–1.67	.15
MMSE	0.90	0.77–1.06	.22
ADL			.09
Score = 20	1		
Score:21–40	1.86	0.46–7.63	.39
Score: 41–60	4.67	0.67–32.30	.12
Score: 61–80	25.31	1.99–322.22	.01
SSRS-utilization of support	0.80	0.60–1.08	.14
Negative LES			<.001
Score: 0–8	1		
Score: 9–32	41.65	8.14–213.12	<.001
Score: >32	226.41	19.87–2579.97	<.001
Constant	0.62		.83

In this model, method = enter, *P* = .962 in Hosmer and Lemeshow test.

ADL = Activity of Daily Living, CI = confidence interval, LES = Life Event Scale, MMSE = Mini-mental Status Examination, NIHSS = National Institutes of Health Stroke Scale, OR = odd ratio, SSRS = Social Support Rating Scale.

**Table 5:** Binary logistic regression analysis of risk factors for PSD (Model III).

	OR	95% CI	*P*
Gender			
Male	1		
Female	1.98	0.76–5.12	.16
History of stroke			
No	1		
Yes	2.88	1.06–7.83	.04
Stroke symptom severity			
Mild (NIHSS score: 0–4)	1		
Moderate (NIHSS score: 5–15)	0.30	0.07–1.29	.11
MMSE	0.93	0.84–1.04	.20
ADL			<.01
Score = 20	1		
Score:21–40	3.54	1.26–9.96	.02
Score: 41–60	5.30	1.34–20.99	.02
Score: 61–80	33.36	5.37–207.12	<.001
SSRS-utilization of support	0.79	0.62–0.99	.04
Constant	3.65		.40

In this model, method = enter, *P* = .879 in Hosmer and Lemeshow test.

ADL = Activity of Daily Living, CI = confidence interval, MMSE = Mini-mental Status Examination, NIHSS = National Institutes of Health Stroke Scale, OR = odd ratio, SSRS = Social Support Rating Scale.

## 4. Discussion

This study determined the relationship between clinical characteristics and PSD in patients with mild or moderate strokes. Indeed, negative life events were independently associated with depression in stroke patients. Other factors, such as gender, stroke frequency, stroke symptom severity, poor ADL ability, cognitive impairment, and utilization of social support, were likely associated with PSD.

Nearly one-third (35.7%) of our sample had depression, and females were more likely to be depressed than males. The findings of the current study were consistent with previous studies; the prevalence rates were similar^[6,7]^ and females were more vulnerable to depression.^[26]^ In contrast with previous studies, age and history of hypertension and diabetes did not influence the incidence of depression in the present study, which indicated that these risk factors for stroke were not associated with PSD. We should pay more attention to stroke features and MDD-related risk factors, although the heterogeneity of results may be due to different population sources.

Among all the characteristics of stroke patients considered in the current study, the impact of negative life events on the prevalence of depression was constant and significant. Negative life events are the cornerstone for causing stress, and plays an important role in the onset of depressive disorders.^[27,28]^ Patients should have access to psychological management resources that can manage negative emotions caused by stress. Women and pessimists have a poorer ability to manage emotions and higher negative LES scores, which increase vulnerability to depression.^[29]^ In the present study, the stroke frequency was independently associated with PSD after eliminating the effect of negative LES scores. This finding suggested that a stroke attack might be a negative life event that impacts the incidence of depression. Interestingly, there were no significant differences in lesion side and site between the NDS and PSD patients. This finding indicated that a cerebral injury in stroke patients does not contribute to depression, and indirectly confirmed that stress and failure to manage emotions leads to depression in stroke patients. Moreover, our findings suggested that the relationship between ADL ability and PSD is also mediated by negative life events. The possible reason for this conclusion is that poor ADL ability produces chronic stress stimulation, which may be one of the driving factors behind the emergence of depression.

Additionally, social support was thought to be a protective factor against depression through emotional support, motivation for treatment, and support with daily functioning.^[14]^ Family support is the most important to some stroke patients who remain dependent on caregivers regarding activities of daily living. Previous studies had showed that social support, including family support, friends’ support and significant others’ support could buffer detrimental effects on depression caused by stressful life events, suggested that social support had a mediating effect on the association between stressful life events and depression.^[30,31]^ Moreover, previous studies have emphasized that the subjective social support rather than objective social support might be crucial for developing PSD^[32–34]^; however, our findings did not demonstrate a significant association between social support and depression. Subjective and objective social support did not differ significantly between NDS and PSD patients. This inconsistency may be due to the study sample. Medical expenses were covered by medical insurance for all study participants and the standard of living in Guilin city is higher than rural areas, which might result in less family pressure and more family support to patients. Therefore, a lack of social support was not a major concern for most stroke patients in our sample; however, PSD patients in the current study had poor utilization of social support. Negative life events had an impact on the independent association between utilization of social support and depression. This finding indicated that utilization of social support was likely protective against depression by reducing the impact of life events on the negative emotions.

Stroke symptom severity was significantly increased in PSD patients compared to NDS patients; however, stroke symptom severity was not independently associated with PSD after eliminating life event factors. In contrast, Chau et al^[35]^ reported that patients with moderate stroke symptoms were nearly twice as likely to develop depression compared to patients with mild symptoms. The heterogeneity of results is likely due to differences in study methods. In general, the severity of stroke symptoms reflects the degree of disability in patients, implying that the stroke symptom severity is closely correlated with ADL ability, and thus the lack of an independent association with PSD is affected by ADL ability. Chau et al^[35]^ did not analyze ADL ability in the logistic regression analysis, which may be the major reason for divergent findings.

Other characteristics were also shown to be associated with depression after a stroke, among which were poor sleep quality and a lack of interest in pleasurable activities were the most predictive. These 2 factors were involved in the diagnosis of depression in the current study, and both were clinical manifestations of depression. Therefore, the close association between these 2 factors and PSD were a natural response. Moreover, PSD patients demonstrated more cognitive deficits than NDS patients, and several patients met the criteria for dementia. A previous study showed that cognitive function might be transiently impaired in patients with an acute stroke, and improved after rehabilitation.^[36,37]^ Thus, the cognitive deficits might be a prominent feature in acute stroke.

## 5. Limitations

Certain limitations and methodologic issues should be considered when interpreting the present findings. First, it might be inappropriate to explore mild and moderate stroke without considering severe stroke, which could result in selection bias. Specifically, most of severe stroke patients could not complete the scale assessment because of a consciousness disorder or severe disability. Therefore, the present study recruited mild and moderate stroke subjects, with NIHSS scores < 16. Second, due to the cross-sectional study design, we could not track changes in depression severity and prevalence over an extended period of time in stroke patients. Thus, longitudinal designs and more accurate diagnosis of PSD should be addressed in future research.

**Figure 1:**
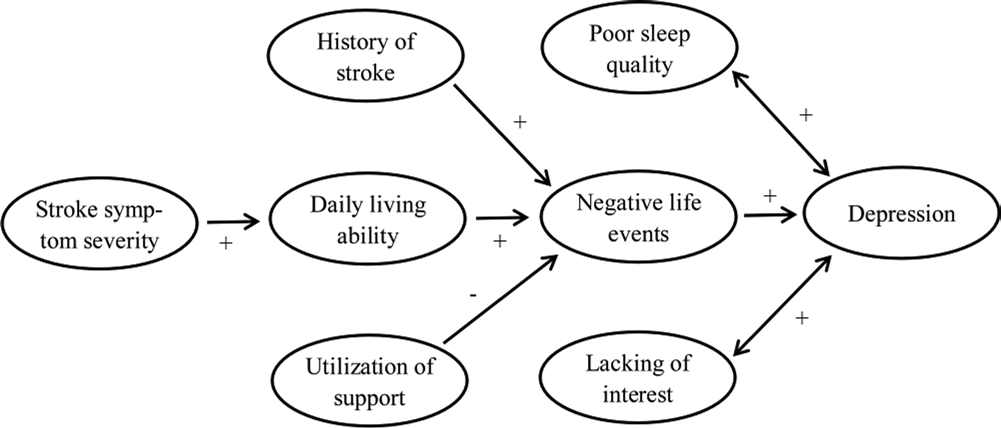
. Possible relationship between risk factors and depression (speculated based on the present results and previous findings).

In summary, negative life events were the major independent predictor of PSD, and likely mediated the correlation between stroke frequency, ADL ability, utilization of social support, and depression in stroke patients. The association between NIHSS scores and PSD might be affected by ADL ability. According to our findings and the previous studies, we speculated a possible relationship among these factors (Fig. 1). The present study warrants further investigations to explore the possible etiopathogenic mechanisms underlying PSD. Clinical intervention for depression might be of vital importance to prevent the developing depression in stroke patients.

## Acknowledgments

We would like to thank all the subjects for their participation in this study, and express our gratitude to International Science Editing (http://www.internationalscienceediting.com) for the expert linguistic services provided.

## Author contributions

**Data curation:** Jing Wu, Junlin Feng, Jingzi Jiang.

**Supervision:** Kaixiang Liu, Hao Li.

**Methodology:** Wenxiang Liao, Jingzi Jiang.

**Writing – original draft:** Wenxiang Liao, Danlei Chen.

**Writing – review & editing:** Wenxiang Liao.
